# Case clusters of Parkinson’s disease

**DOI:** 10.1016/j.prdoa.2023.100211

**Published:** 2023-07-12

**Authors:** E. Fuller Torrey, Judy Miller, Ann Bowler

**Affiliations:** Stanley Medical Research Institute, 9800 Medical Center, Suite C-050Rockville, MD 20850, United States; Stanley Medical Research Institute, retired, United States

To the editor:

Most non-genetic case clusters of Parkinson's disease occur in industrial settings when several individuals are exposed to a known Parkinson's risk factor such as heavy metals [1]. Case clusters among individuals who have shared work or living spaces but do not have a common exposure to a known Parkinson's risk factor are relatively uncommon and often suspected of being coincidental [2], [3], [4]. Here we report a case cluster of the latter type among three individuals, the authors, who variously worked together over 24 years in a medical research unit.

**Case 1:** Male physician, directed the research unit from ages 51 to 75. At age 74 noted a unilateral resting tremor and self-diagnosed Parkinson's disease, confirmed by movement disorders specialist two months later. Now age 85, lives at home with family member.

**Case 2:** Female research assistant, worked in the research unit for 16 years from age 47 to 63. At age 67 noted deterioration of handwriting, unilateral arm tremor and decrease in arm swinging when walking. At age 68 diagnosed with Parkinson's disease by movement disorders specialist. Now age 74, lives at home with family member. Worked closely with case 1 for 16 years and with case 3 for two years.

**Case 3:** Female biostatistician, worked in the research unit for 10 years from ages 53 to 63. At age 80 was noted to have a shuffling gait followed by falls two years later. Was then diagnosed with Parkinson's disease, confirmed by a movement disorders specialist at age 85. Now age 87, lives in an assisted living facility. Worked closely with case 1 for 10 years and with case 2 for two years.

None of the three had a family history of Parkinson's disease. The medical research unit, which focused on twin and epidemiological studies, included six other full-time staff, none of whom have become affected. The unit members had shared and contiguous offices initially in a hospital research building with five other medical research units. The senior author has maintained contact with individuals who were in four of the other units with no known cases of Parkinson's disease. Later this research unit moved to a commercial office building. Over the 24 years there was no known exposure to heavy metals, pesticides or other chemicals and both buildings used the public water supply. So far no family member of the three affected individuals has developed Parkinsonian symptoms.

Since there is no known common exposure among the three individuals to a Parkinson's risk factor, we conclude that this case cluster is most likely coincidental. However we believe that such case clusters should be reported and carefully examined for common risk factors.

## Declaration of Competing Interest

The authors declare that they have no known competing financial interests or personal relationships that could have appeared to influence the work reported in this paper.
